# Concurrent Visualization of Acoustic Radiation Force Displacement and Shear Wave Propagation with 7T MRI

**DOI:** 10.1371/journal.pone.0139667

**Published:** 2015-10-06

**Authors:** Yu Liu, Brett Z. Fite, Lisa M. Mahakian, Sarah M. Johnson, Benoit Larrat, Erik Dumont, Katherine W. Ferrara

**Affiliations:** 1 Department of Biomedical Engineering, University of California Davis, Davis, CA, 95616, United States of America; 2 UNité d’Imagerie par Résonance Magnétique et Spectroscopie, NeuroSpin, CEA, Gif Sur Yvette, France; 3 Image Guided Therapy, Pessac, France; Rensselaer Polytechnic Institute, UNITED STATES

## Abstract

Manual palpation is a common and very informative diagnostic tool based on estimation of changes in the stiffness of tissues that result from pathology. In the case of a small lesion or a lesion that is located deep within the body, it is difficult for changes in mechanical properties of tissue to be detected or evaluated via palpation. Furthermore, palpation is non-quantitative and cannot be used to localize the lesion. Magnetic Resonance-guided Focused Ultrasound (MRgFUS) can also be used to evaluate the properties of biological tissues non-invasively. In this study, an MRgFUS system combines high field (7T) MR and 3 MHz focused ultrasound to provide high resolution MR imaging and a small ultrasonic interrogation region (~0.5 x 0.5 x 2 mm), as compared with current clinical systems. MR-Acoustic Radiation Force Imaging (MR-ARFI) provides a reliable and efficient method for beam localization by detecting micron-scale displacements induced by ultrasound mechanical forces. The first aim of this study is to develop a sequence that can concurrently quantify acoustic radiation force displacements and image the resulting transient shear wave. Our motivation in combining these two measurements is to develop a technique that can rapidly provide both ARFI and shear wave velocity estimation data, making it suitable for use in interventional radiology. Secondly, we validate this sequence *in vivo* by estimating the displacement before and after high intensity focused ultrasound (HIFU) ablation, and we validate the shear wave velocity *in vitro* using tissue-mimicking gelatin and tofu phantoms. Such rapid acquisitions are especially useful in interventional radiology applications where minimizing scan time is highly desirable.

## Introduction

Tissue properties, including stiffness, change in many disease states. Malignant tissue is typically stiffer than the healthy surrounding tissue, and this difference in stiffness results in a local change in the shear wave speed. There has been great interest in developing techniques to create 2D and 3D maps of the shear modulus, and numerous elastographic techniques have been developed to accomplish this goal.

Magnetic Resonance Elastography (MRE), first introduced in 1995, is a 3D technique to measure tissue stiffness non-invasively [1, 2]. An external vibrator generates a steady state shear wave in the organ of interest. A motion sensitized MR sequence is synchronized with the external vibrator and used to record the propagating shear wave in the phase of the MR signal. In the last 20 years, researchers have investigated and employed MRE for mapping the stiffness of various soft tissues: brain [3–6], breast [7–10], heart [11, 12], lung [13], muscle [14], liver [15–22], spleen [23], kidneys [24] and pancreas [1]. A disadvantage of MRE is that it requires an external mechanical vibrator [25, 26] to generate the shear wave, a complex and sometimes patient-unfriendly additional hardware. Furthermore, induced shear waves have poor penetration in deep organs.

In 1998, Sarvazyan et al. proposed a similar motion sensitized sequence to image the displacements induced by a focused ultrasound beam in a phantom [27]. Although the magnitude of these displacements is rather small (on the order of 10 μm), they can be measured with MR by encoding them in the phase of the MR signal using additional magnetic field gradients, timed to correspond with the displacement. Based on this principle, Magnetic Resonance Acoustic Radiation Force Imaging (MR-ARFI) was developed by McDannold and Maier [28] using a pair of Stejskal-Tanner gradients (unipolar gradients) as motion-sensitizing gradients. Later, Chen et al. [29] proposed a modified spin echo MR-ARFI sequence, where the unipolar gradients were replaced by a pair of inverted bipolar gradients in order to improve the sensitivity to micron-scale displacement without decreasing the signal-to-noise ratio (SNR). Larrat et al. first used MR-ARFI *in vivo* for the guidance of high intensity focused ultrasound (HIFU) in rat brain [30]. The bipolar gradient method, with its robustness against bulk motion and background phase distortion, was shown to increase SNR by approximately 2.5-fold over the unipolar gradient method under identical conditions. Images were further improved by reducing diffusion-weighting and optimizing the encoding pulse width [29].

Concurrently, Souchon *et al*. proposed using repeated MR-ARFI acquisitions with progressive time shifts to catch the propagation of the transient shear wave induced by the radiation force [31]. This so-called “transient MRE” technique presents the advantage of inducing large wave amplitude in deep tissues although it still requires an external apparatus to generate it. In addition, MRE requires one to solve an ill-posed inverse problem to calculate the local stiffness whereas changes in displacement at focus and shear wave speed can be directly measured using MR-ARFI, similar to ultrasound based transient elastography techniques [32–38].

The uncertainty in the ARFI signal encoded into the phase image is inversely proportional to the magnitude SNR [39]. The quasi-linear gain in SNR with magnetic (B_0_) field strength can be exploited to gain both higher spatial and phase resolution. Moreover, the increased precision of the phase maps at high field permits the full exploitation of high performance gradient systems. The magnitude of the change in phase for a given displacement is proportional to the integrated area of the gradient waveform. Gradient systems with high maximum amplitude and slew rates can thus be used to encode a displacement more rapidly, while the increased phase precision at higher field ensures that displacements are decoded more reliably. Therefore, the increase in sensitivity to displacement detection with increasing field strength promises an improvement in image quality and precision.

In applying MR-ARFI to small animal imaging studies, a small focal size (ie. high ultrasound frequency) is desirable. The combination of a 7T MR scanner equipped with strong gradients (200 mT/m) and 3 MHz Focused Ultrasound (FUS) is advantageous as it can perform dual functions: simultaneous accurate measurement of tissue response to radiation force and accurate beam localization as a result of the high image resolution. We extend this further by incorporating a measurement of transient shear wave speed within the same sequence.

At high field and with high performance gradients, the higher SNR and displacement sensitivity can be exploited to create a modified MR-ARFI sequence based on the inverted bipolar Motion Encoding Gradient (MEG) scheme. This modified scheme is able, with the second pair of MEGs, to image the shear wave generated by the application of ultrasound during the previous pair of MEGs. To reliably image the shear wave, the duration of the MEGs must be dramatically reduced (from >10 ms to ~1ms) from typical MR-ARFI implementations. While this does entail deviating from an optimal ARFI motion encoding scheme, the losses in SNR do not significantly compromise image quality at higher field strength.

An important application of such technology is the assessment of changes in the mechanical properties of tissue with treatment. Multiple studies have proven that reversible or permanent elasticity changes occur after tissue ablation [40–42]. Our goal is to demonstrate a new method to monitor the success of HIFU therapy by localizing the beam and measuring both the displacement at the focus and the shear wave velocity. The combination of multiple measurements in a single sequence reduces the scan time allowing such a sequence to be used during therapeutic ultrasound applications. Such a sequence can be applied between ablative pulses to assess the extent of treatment and to plan the next insonation. For this purpose, we will investigate the use of the modified MR-ARFI sequence in two ways. We will first measure the ARFI signal at the focus and estimate the corresponding displacement. For a given acoustic power and sequence, the displacement is larger with a softer medium. Second, we will measure the distance of propagation of the shear wave from the focal spot between the two MEG. From this, we will deduce a shear wave speed and thus further characterize the tissue mechanical properties.

## Material and Methods

### MR guided Focused Ultrasound System (FUS) and measurement overview

**Fig 1:**
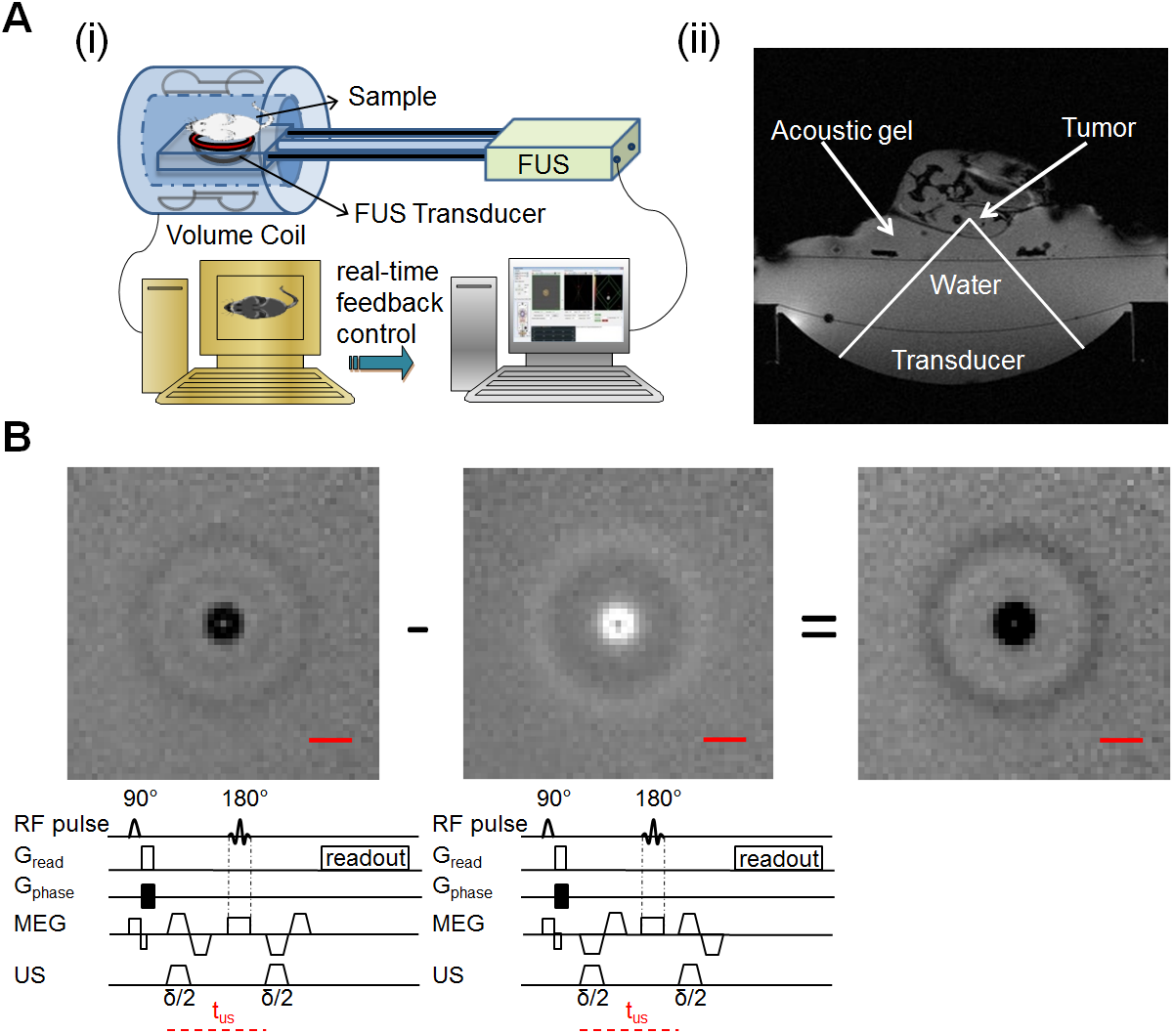
Overview of the experiments. (Ai) Schematic diagram of MR-guided focused ultrasound system (MRgFUS). The transducer is a 16-element annular array with focal spot size of 0.5 x 0.5 x 2 mm^3^. (Aii) MRI axial magnitude image of the experimental setup, demonstrating: the location of the transducer; water bath linking the motorized transducer to a water-tight membrane; acoustic gel above the membrane; and tumor and surrounding tissue. (B) The motion encoding sequence used to acquire both the acoustic radiation force displacement and the shear wave propagation. The illustrated sequence was acquired with both positive and negative bipolar motion encoding gradients (MEGs). The sonication duration (δ/2) coincided with the first half of each bipolar gradient period. The images were acquired in an 8% gelatin phantom, where subtracting the image with negative MEG polarity (middle) from the positive image (left) results in the final phase image (right). The scale bar in the images represents 8 mm.

A prototype MR-guided focused ultrasound system (Image Guided Therapy, Pessac, France) was used in this study. A schematic diagram of the system is shown in Fig 1A(i). Here, the images were acquired by a 7 T MR scanner (Biospec 70/30 USR, Bruker Biospin, Germany) utilizing a radiofrequency (RF) coil with internal diameter of 154 mm for both transmitting and receiving and a B-GA-20S gradient system (200 mT/m maximum amplitude, 640 T/m/s slew rate; Bruker Biospin, Ettlingen Germany). The MR system is running under ParaVision 5.1. A typical MR axial magnitude image obtained with the experimental system is shown in Fig 1A(ii).

The MR-compatible ultrasound transducer consists of a 16-element annular array (IMASONIC SAS, France, 3 MHz central frequency, 300 kHz bandwidth, 256 W maximum electric power, acoustic efficiency > 65%, 48 mm diameter, 35 mm radius of curvature, adjustable focusing depth, 0.5 × 0.5 × 2 mm^3^ focal spot volume at -6 dB) and an embedded MR compatible 2-dimensional positioning system.

Data were received in real time by a separate multi-core PC running the IGT software Thermoguide. We performed online phase unwrapping, image reconstruction and measurement of temperature elevation. This provides us with real-time (subsecond) control of each aspect of the signal processing and thermometry. The use of the system to control hyperthermia has been published [43]. We have demonstrated thermometry frame updates in <0.25 s and temperature accuracy of <1°C.

The FUS beam profile was measured with an Onda fiber optic hydrophone (Onda Corporation, Sunnyvale, CA), which has 150 μm active diameter. The hydrophone probe was mounted on a three-dimensional positioning system (Newport motion controller ESP 300, Newport 443 series), which has a minimum step size of 0.1 μm and a maximum scan range of 50 mm. The hydrophone was connected to an oscilloscope (sampled at 100 MHz) where the received voltage was recorded and then converted off-line to pressure based on the Fresnel formula. The transducer, immersed in a degassed water tank, emitted a 10-μs short pulse to eliminate standing waves and minimize the temperature increase and cavitation activity. The hydrophone was scanned in both lateral and axial direction at the natural focal plane and the normalized FUS beam profile was calculated.

All measurements are reported as the mean and standard deviation of the measurement.

### MRI acquisition sequence

MR images were acquired using a modified spin-echo imaging sequence, shown in Fig 1B. The inverted bipolar motion encoding gradient (MEG), can be incorporated in any orientation. In this study, the ultrasound beam and radiation force were oriented in the vertical direction and the MEG was perpendicular to the US beam in the coronal direction. A trigger generated from the MR imaging sequence was sent to the focused ultrasound electronics which synchronized the ultrasound pulses with the gradient waveforms. In each repetition, the US was turned on during the first half of each MEG period. The residual phase map was obtained after the subtraction of a pair of phase maps acquired with inverse MEG polarity, which can increase the SNR and also eliminate the bulk motion and background variations.

The US induced displacement, d, is encoded in the phase, *Φ*, of the MR image according to the following equation:

$$\varphi =\gamma \int_{T_{0}}^{T_{0}+\delta }\vec{G}(t)•\vec{d}(t)dt$$ (1)

where, γ is the gyromagnetic ratio of ^1^H, *G(t)* is the amplitude of the motion encoding gradient at time t, *d(t)* is the displacement at time *t*, *T*_*0*_ is the start time and *δ* is the duration of the encoding gradient. If the shape of the encoding gradients can be approximated as rectangular and the displacement remains constant for the time δ the encoding gradients are applied, then the integral can be approximated as the product of the gradient strength, G, the encoding time, *δ*, and the amplitude of displacement along the axis of the encoding gradient, d. The displacement can then be calculated as follows:

$$d=\frac{\Delta \varphi }{2\gamma G\delta }$$ (2)

where *ΔΦ* represents the phase difference between two phase images acquired with opposite encoding gradient polarities. For trapezoidal gradients, as were used in this study, approximating the gradient waveform as a rectangle is a reasonable approximation when the rise time is small compared to the duration of the gradient application. For a 3.5 ms MEG duration, the error introduced by the trapezoidal approximation should be less than 3% in our case due to our high slew rate. The use of (2) likely underestimates the true displacement due to the timing of the tissue response relative to the first and second gradient lobes and the finite slew rate as described above.

### Tissue mimicking phantom materials

As in many ultrasound studies of tissue mechanical properties, tissue mimicking viscoelastic materials are used in this study. According to [44], the calculated shear wave speed in breast fatty tissue is 1.7 m/s, in breast parenchyma is 3.1–3.2 m/s and in invasive ductal carcinoma is 6.6 m/s [44]. The gelatin phantoms and tofu used in this study provide shear wave velocities that are expected to span a range from below 1.7 m/s to ~4 m/s. Silken tofu provides a homogeneous material with a shear wave velocity greater than standard gelatin materials and a higher MR signal to noise ratio than gelatin phantoms and is used here in comparison studies. For our typical MRI acquisition parameters, the signal to noise ratio for the 8 and 10% gelatin phantoms are 68 and 80% of that for the tofu phantom, respectively. A set of gelatin phantoms with bovine skin gelatin powder (Sigma Co., St. Louis, USA) concentrations from 5 to 15% was also used to measure the ultrasound beam profile and to determine the effect of phantom properties on the estimates of displacement and shear wave velocity. The 8% gelatin phantom was chosen as the default for other studies (e.g. beam localization within the MRI environment) as it has a shear wave velocity of 1.7 m/s that corresponds with tissues such as fatty breast tissue. Such gelatin phantoms have been widely used as a tissue mimicking model in *in vitro* studies due to the similarity between the phantom properties and soft tissue [45]. The gelatin powder was mixed with degassed deionized water at ambient temperature, heated until all powder was dissolved and the resulting mixture was poured into a mold.

### Beam localization and MR-ARFI *in vitro*

The relationship between ultrasound pressure and the displacement at the focus was first measured in an 8% gelatin phantom using a spin-echo sequence modified to include motion-encoding gradients along the direction of ultrasound propagation (multiple spin multiple echo (MSME); repetition time (TR)/ echo time (TE)/ flip angle (FA) = 500 ms/25.8 ms/90°; refocusing angle: 180°; MEG = 140 mT/m; field of view (FOV) = 80 x 80 mm^2^; matrix (MTX) = 240 x 240; slice thickness (ST) = 1 mm, 1 slice, number of excitations/averages (NEX) = 1; bandwidth (BW) = 27 kHz). The applied ultrasound peak negative pressure ranged from 3.2 to 12.2 MPa using two 3.5 ms pulses with t_us_ (as defined in Fig 1B) of 10 ms.

The temperature elevation induced by a series of the pulsed sonications used for these measurements was estimated by MR thermometry (proton resonance frequency shift method). A function generator (Agilent 33500B) triggered the ultrasound with the same duty cycle as the MRI sequence. The MR scanner acquired a series of gradient echo images (FLASH; TR/TE/FA = 15.6ms/8.0ms/15°; FOV: 88 x 88 mm^2^; MTX = 96 x 96; ST = 1 mm, 1 slice; BW: 27kHz) and sent the data to Thermoguide in real time where it was reconstructed into phase and magnitude images. The phase reconstructed images were used to generate temperature difference maps in real time [30]. In order to mimic the ARFI parameters during temperature measurement, two ultrasound bursts of 3.5 ms (with t_us_ of 10 ms) were emitted with 18.8 Watts acoustic power (9.1 MPa peak negative pressure). The temperature was estimated based both on one pair of ultrasound pulses and ultrasound pulsing repeated 96 times with 200 ms repetition time.

### Ultrasound beam profile assessed at 7T MRI

Ultrasound beam profile measurements were acquired in an 8% gelatin phantom using multi-slice phase reconstructed images of the coronal planes (orthogonal to the direction of ultrasound propagation) were acquired (MSME; TR/TE/FA = 3500 ms/25.8 ms/90⁰; refocusing angle: 180°; FOV = 80 × 80 mm^2^; MTX = 240 × 240; ST/SI = 1 mm/1 mm, 7 slices) at a -3, -2, -1, 0, 1, 2 and 3 mm distance from the geometric focus of the transducer along the transducer axis. The ultrasound parameters for each TR were: 29.4 Watts acoustic power (10.6 MPa peak negative pressure) and 7 ms duration (two 3.5 ms pulses with t_us_ of 10 ms). The diameter of the area enclosed within a -3 dB contour line and the peak amplitude have been calculated at each depth.

### Evaluation of ARFI signals as a function of gelatin concentration

To evaluate the ability of our methods to distinguish varying mechanical properties, gelatin phantoms were made from various bovine skin gelatin powder concentrations and ARFI signals measured with constant acoustic power. The displacements were then calculated off-line using Eq (2) and compared. The samples were imaged with a modified spin-echo sequence (MSME; TR/TE/FA = 200 ms/7.5 ms/90°; refocusing angle = 180°; FOV = 64 x 64 mm^2^; MTX = 64 x 64, ST = 1 mm, 1 slice; NEX = 1, MEG = 120 mT/m gradient amplitude) and insonated with 5.7 acoustic Watts (4.35 MPa peak negative pressure) and 10 ms pulse duration (two 5 ms pulses with t_us_ of 13 ms).

### Simulation of physiological breathing motion in the phantom system

MR-ARFI performance in the presence of artificial motion was tested in gelatin phantoms using an MR-compatible experimental motion simulator. The simulator consisted of a linear motor, PVC pipe sealed with plastic wrap and a plastic bottle. The bottle and the pipe were connected with a piece of tubing (ID: 1/8”, OD: 1/4”) and filled with degassed water. A metal bar driven by a linear motor periodically compressed the water bottle, which generated sinusoidal motion at the other end of the membrane with a duty cycle of 5%. The period of motion was adjusted within the range of 3–4 s/cycle. The phantom was placed on top of the pipe membrane while motion was contained in the vertical plane (which was parallel to the ultrasound beam propagation direction). The motion of the membrane was monitored by an MR-compatible small animal monitoring and gating system (Model 1025, SA Instruments, Stony Brook, NY, USA). The amplitude of the periodic motion ranged from 2 to 5 mm depending on the pressure applied at the bottle end. The ARFI signals acquired with applied sinusoidal motion were then compared with those measured without motion. As above, the samples were imaged with the modified spin-echo sequence (MSME; TR/TE/FA = 200 ms/13.5 ms/90°; refocusing angle = 180°, FOV = 64 x 64 mm^2^; MTX = 64 x 64; ST = 1 mm, 1 slice; 120 mT/m MEG gradient amplitude) and insonated with 5.7 acoustic Watts (4.35 MPa peak negative pressure) and 10 ms pulse duration (two 5 ms pulses with t_us_ of 13 ms).

### Shear wave propagation and its velocity estimation *in vitro*

In addition to the sequence shown in Fig 1B and to further investigate the nature and source of the radial waves observed in phantoms following insonation, a modified sequence (MSME; TR/TE/FA = 200 ms/23.5 ms/90°; refocusing angle = 180°, FOV = 85 x 85 mm^2^; MTX = 96 x 96; ST = 1 mm, 1 slice; 140 mT/m MEG gradient amplitude), lacking the second MEG lobe, was used to image a 80 mm disc-shaped 8% gelatin phantom using the following ultrasound parameters: 18.8 acoustic Watts (9.1 MPa peak negative pressure), 7 ms total duration with t_us_ = 10 ms. The results were compared with results obtained from the original sequence in Fig 1B (ie. using 2 bipolar MEG).

In order to estimate the shear wave speed, an add-in delay, t, was programmed into the sequence, before and after the 180° refocusing pulse and between the two MEGs. As the delay was increased, we expected the shear wave to propagate further from the ultrasound beam center. Phase images were acquired for t = 0, 1, 2 and 4 ms (corresponding to an absolute shear wave propagation time of 10, 12, 14 and 18 ms) (MSME; TR/TE/FA = 200 ms/23.5 ms/90°; refocusing angle = 180°, FOV = 85 x 85 mm^2^; MTX = 96 x 96; ST = 1 mm, 1 slice; 140 mT/m MEG gradient amplitude). For the shear wave visualization studies, the acoustic power was increased: here, the acoustic power was 18.8 Watts (9.1 MPa peak negative pressure) and the duration was shortened to 2 ms with t_us_ = 5 ms in order to improve visualization.

In order to accurately determine the position of the ultrasound beam focus in the phase image, a longer duration (7 ms ultrasound pulse) ARFI image was first acquired prior to running the modified (2 ms) sequence. Locations of constant phase of the propagating shear wave (radius r) extending outwards from the focus (360° sweep with a 1° increment) were plotted by detecting the negative extrema in the propagating wave in each radial direction, approximating each point to the Cartesian image grid and averaging this result over estimates acquired over 360°. Based on this distance and corresponding propagation time, the propagation speed of the shear wave was calculated. The shear wave speed was then estimated in 8 and 10% gelatin phantoms and in a Silken tofu (firm) phantom.

### *In vivo* MR-ARFI studies

All animal studies were performed under a protocol approved by the Institutional Animal Care and Use Committee (IACUC) of the University of California, Davis. A total of 3 female *neu* deletion (NDL)-tumor bearing mice [46, 47] (7 weeks old, 15–25 g, Charles River, MA) were used for the *in vivo* studies. Anesthesia was induced with 3.5% isoflurane and maintained at 2% isoflurane. Buprenorphine was administered (0.05–0.1 mg/kg subcutaneously) immediately prior to ablation for analgesia. The body temperature was monitored using an MR compatible small animal monitoring system (Model 1025, SA Instruments Inc., Stony Brook, NY). Animals were placed on top of a piece of agarose gel pad in the prone position, such that the tumor could be easily accessed from the side with ultrasound without sonicating other vital organs. The tumor was coupled to the gel pad through ultrasound gel. In Fig 1A(ii), the tumor location was indicated by the red dashed circle. A single ARFI measurement was acquired inside the tumor (MSME; TR/TE/FA = 200 ms/23.5 ms/90°; refocusing angle = 180°, FOV = 64 x 64 mm^2^; MTX = 128 x 128; ST = 1 mm, 1 slice; 140 mT/m MEG gradient amplitude) with ultrasound parameters of 29.4 acoustic Watts (10.7 MPa peak negative pressure), 7 ms duration with t_us_ = 10 ms. The same locations were then thermally ablated using HIFU with the following sonication parameters: 10.6 acoustic Watts (peak negative pressure = 7.7 MPa) and 7 seconds duration. Forty-eight hours post ablation, a second ARFI measurement was acquired inside the tumor. Animals were sacrificed by cervical dislocation. Displacement estimates before and after ablation were then compared.

## Results

### FUS beam profile and ARFI validation

**Fig 2:**
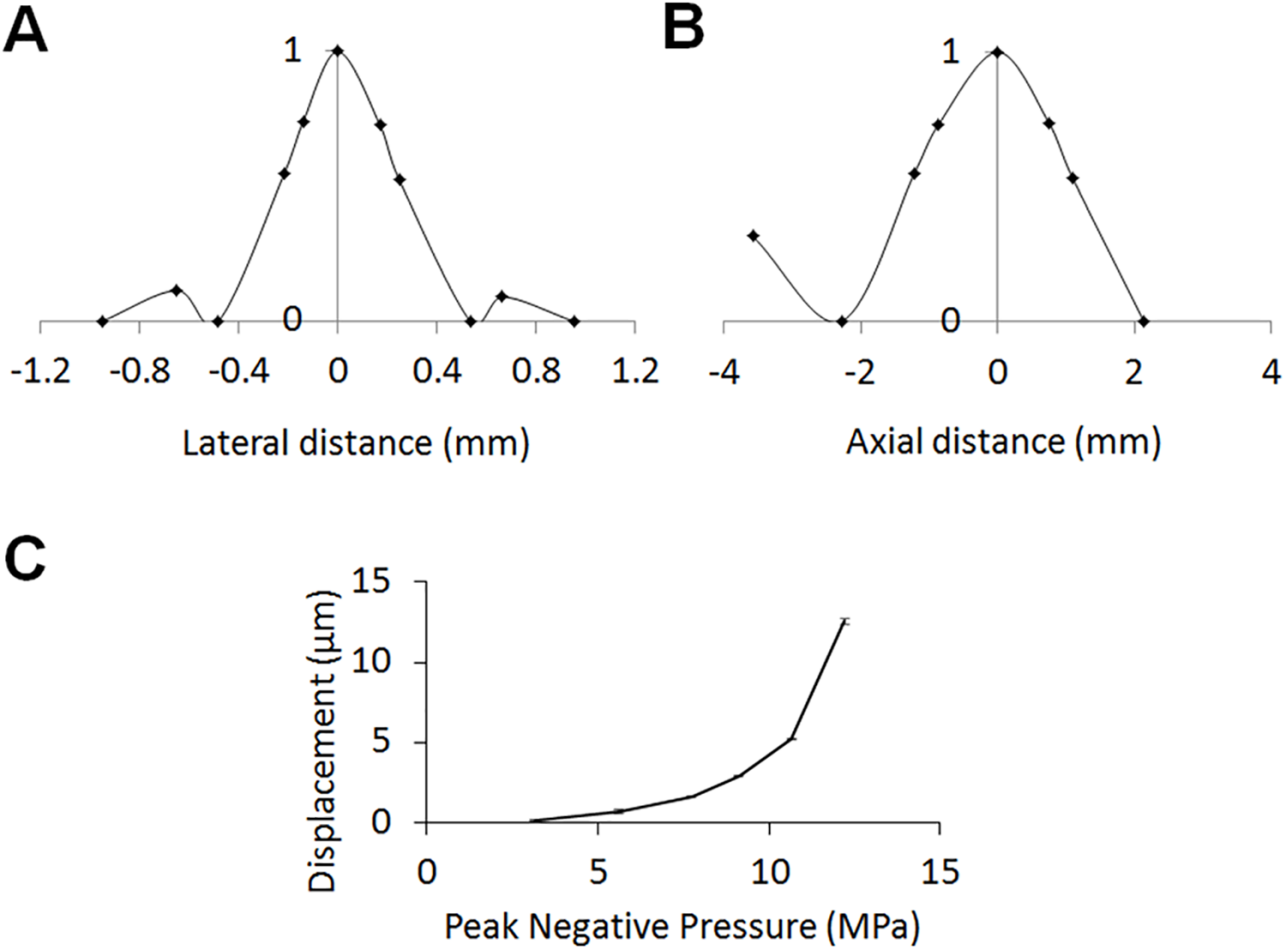
FUS beam profile measurement and ARFI validation. The normalized FUS beam profile in the lateral (A) and axial (B) directions. (C) The displacement versus ultrasound pressure in a bovine gelatin phantom where the peak negative pressure was varied from 3.2 to 12.2 MPa.

The -6 dB beam width in the focal plane was 0.5 mm in the lateral and 2.5 mm in the axial direction (Fig 2A and 2B). The first side lobe occurred ~0.7 mm from the center with an intensity of −10 dB compared to the focal spot.

To verify the MR-ARFI performance, ultrasound was applied to an 8% gelatin phantom, and the relationship between the peak negative pressure applied (from 3.2 to 12.2 MPa) and the displacement was quantified (Fig 2C). As predicted by theory, this relationship is quadratic. For the parameters and sequence used here, the detected displacement ranged from less than 1 to more than 13 μm.

The temperature elevation was estimated by MR thermometry. For a single ultrasound pulse, the change in temperature was not detected by MR thermometry. For a train of 96 pulse pairs at a 200 ms interval (18.8 acoustic Watts, 9.1 MPa peak negative pressure, 7.0 ms duration, t_us_ = 10 ms), the local temperature increased by ~1.5°C.

### Beam localization

**Fig 3:**
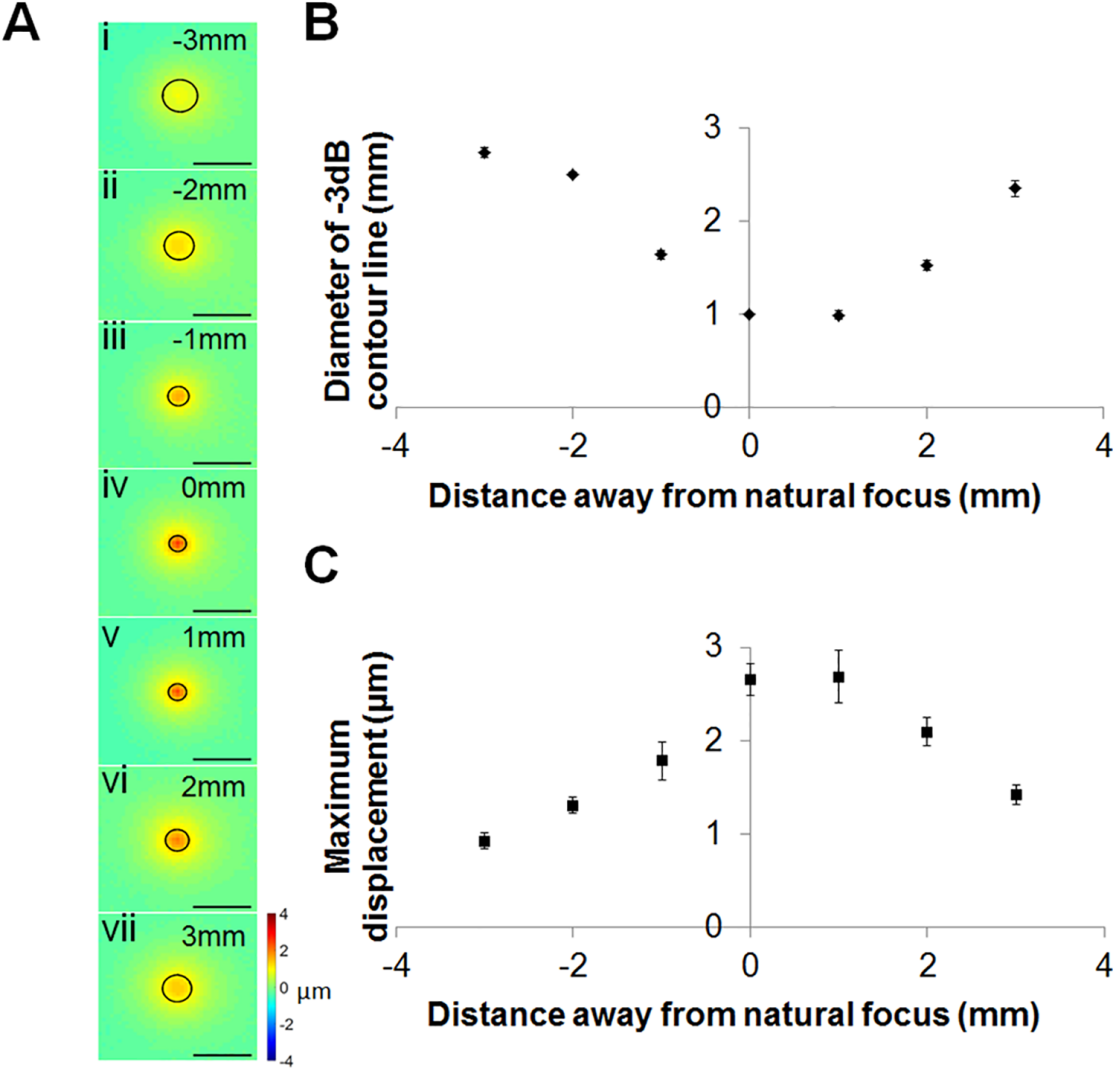
Localization of the acoustic beam by MR-ARFI. (A) Seven coronal slices were acquired at -3, -2, -1, 0, 1, 2 and 3 mm away from the US focal plane along the depth axis with -3 dB contour lines. (B) The diameter of the area enclosed within a -3 dB contour line was calculated at different depths. (C) The peak displacement amplitude at each depth.

The displacement produced by the ultrasound beam was assessed based on the ARFI images acquired while sonicating the 8% gelatin phantom. The diameter of the region enclosed within a -3 dB displacement contour was 2.74 ± 0.05, 2.50 ± 0.02, 1.64 ± 0.04, 1.00 ± 0.00, 1.00 ± 0.05, 1.53 ± 0.05 and 2.35 ± 0.08 mm^2^ at -3, -2, -1, 0, 1, 2, 3 mm from the ultrasound focus, respectively, as aligned with the MR coordinates (Fig 3Ai–Fig 3Avii), Fig 3B). The maximum displacement was 0.96 μm at a depth of -3 mm relative to the focus, increased to 2.65 μm at the focal center and then decreased to 1.42 μm at 3 mm from the focus (Fig 3C). As anticipated, with increasing depth the diameter of the effective displacement narrowed at the transducer focus, and widened again in the far field.

### Effect of physiological motion on displacement estimates

**Fig 4:**
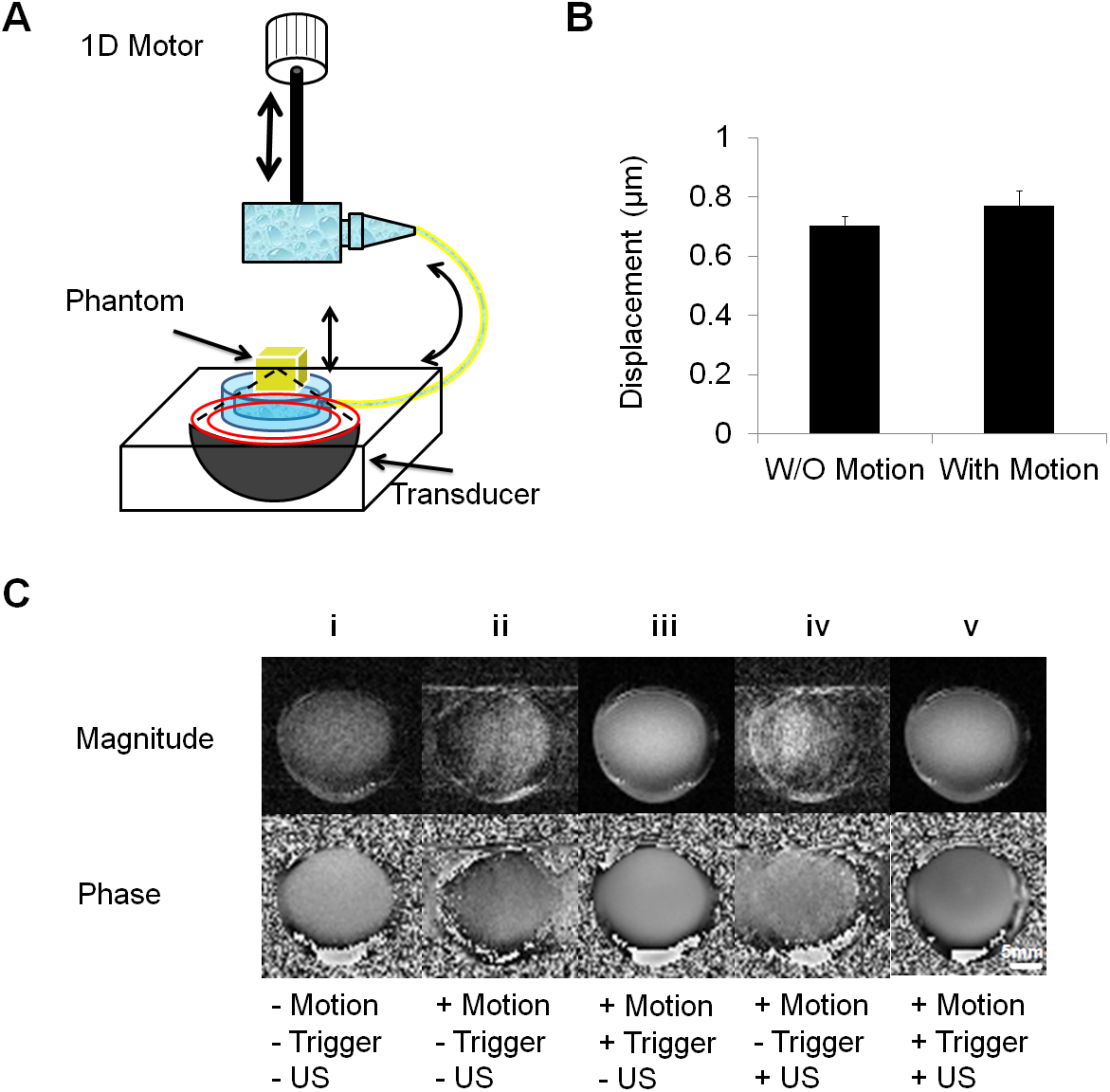
Effect of physiological breathing motion simulator on displacement estimation. (A) Schematic diagram of the MR-compatible respiratory motion simulator. A 1d linear motor is used to periodically depress a water bottle, which generates sinusoidal motion at a membrane connected to the bottle via plastic tubing. The phantom resting on the membrane is thus raised and lowered by the water pressure. The system generates 0.3 Hz sinusoid motion with an amplitude of 4 mm. (B) Comparison of the displacement with and without artificial sinusoidal motion. (C) Comparison of image quality of bovine gelatin phantom with and without motion, sonication and trigger. Magnitude and phase images are shown on the top and bottom row. Column (i) as a control, indicates the image quality without artificial motion. After motion was induced, images were acquired without (column (ii, iv)) and with the trigger (iii, v).

Artificial motion with an amplitude range of 2 to 5 mm was added to mimic respiratory motion and to evaluate the effect of an image gating system with the experimental system (Fig 4A). The estimated displacement at the focus with added artificial motion but without image triggering and gating was 0.77 ± 0.05 μm, which was similar to that measured without motion, 0.70 ± 0.03 μm (Fig 4B). Without motion, the phantom was clearly visualized (Fig 4C(i)). After adding artificial respiratory motion, without the gating system, the images were blurred and distorted, as demonstrated in Fig 4C(ii) (without ultrasound) and 4C(iv) (with ultrasound). Under similar conditions but triggering the acquisition and gating the resulting images (turning acquisition on and off at a specific breathing phase), the effect of motion was suppressed (Fig 4C(iii) and Fig 4C(v)). Therefore, we concluded that our methods were capable of estimating ARF-induced displacement in the presence of physiological motion.

### MR-ARFI in gelatin phantoms and *in vivo* studies

**Fig 5:**
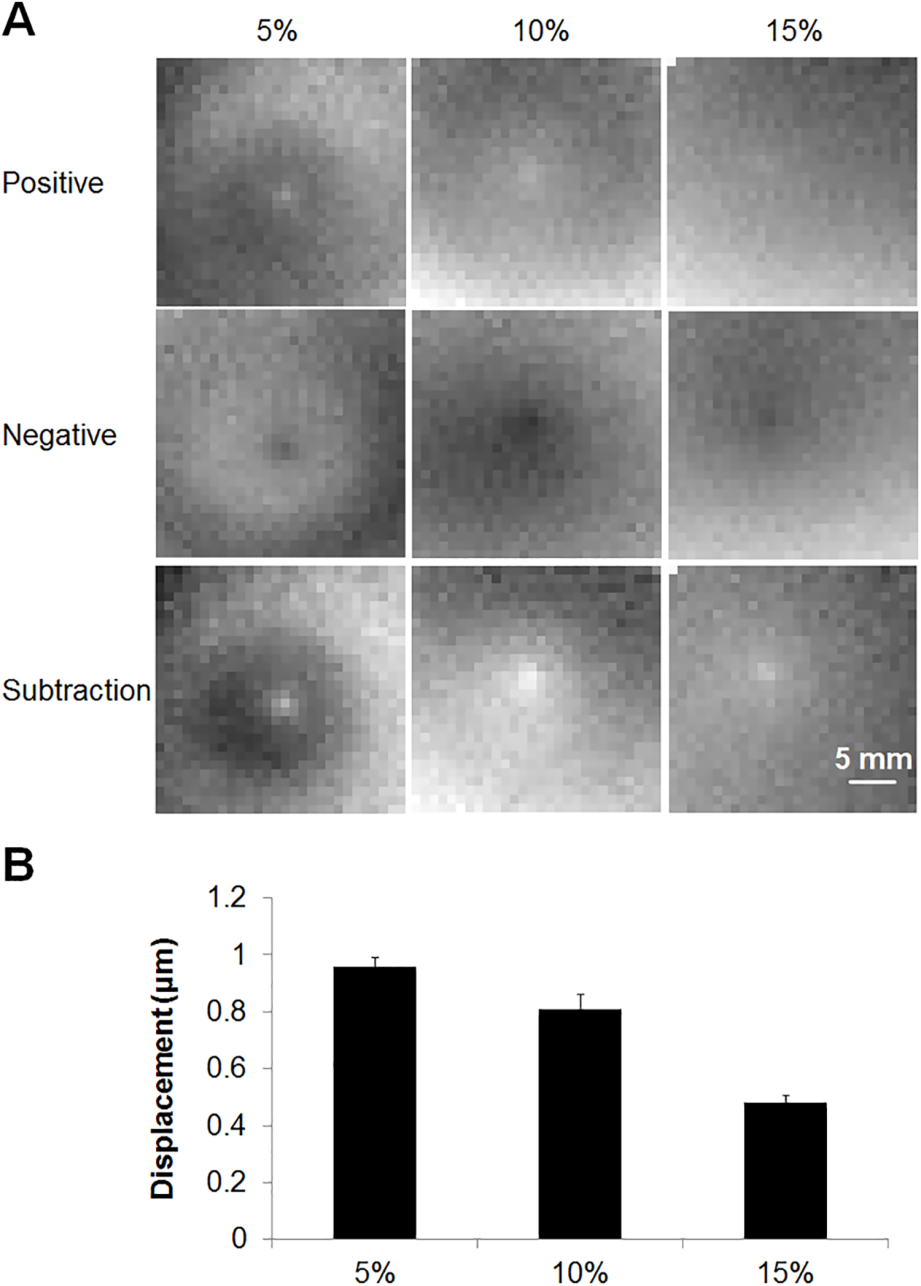
MR-ARFI displacement as a function of gelatin phantom properties. (A) MR-ARFI was tested in gelatin phantoms with 3 different concentrations, 5% (first column), 10% (second column) and 15% (third column). The images acquired with negative MEG polarity (second row) were subtracted from images acquired with positive MEG polarity (first row) to yield the final phase images (third row). (B) The calculated displacement was 0.96 ± 0.03, 0.81 ± 0.05 and 0.48 ± 0.03 μm in 5, 10 and 15% gelatin phantoms, respectively.

MR-ARF-induced displacement was detected in gelatin phantoms with 3 different concentrations: 5% (first column), 10% (second column) and 15% (third column) (Fig 5A) where the negative images (second row) were subtracted from the positive images (first row) resulting in the final phase images (third row). Using the 5.7 W acoustic power and 10 ms pulse duration excitation, the average displacement in the 5, 10 and 15% gelatin phantom was 0.96, 0.81 and 0.48 μm, respectively (Fig 5B) and was easily visualized with MRI.

**Fig 6:**
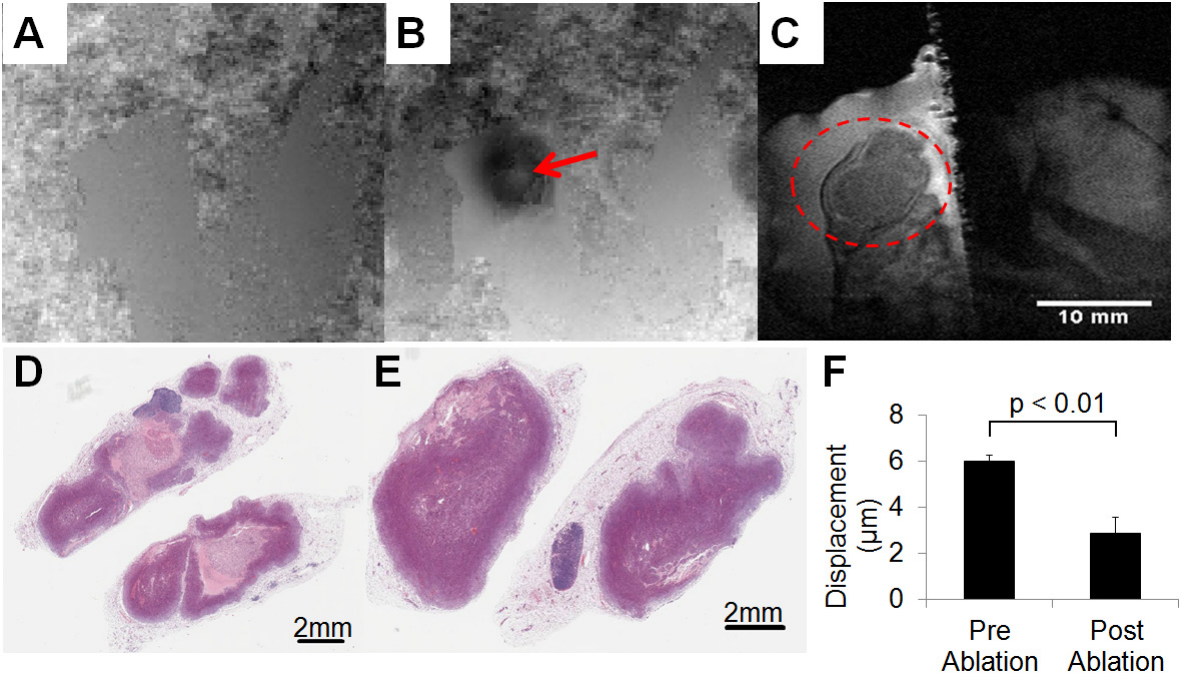
Example of *in vivo* MR-ARFI imaging. Phase images without (A) and with (B) FUS-induced displacement, where the red arrow indicates the location of focus. (C) Magnitude image, where the red circle indicates the tumor region. Corresponding H&E of the treated (D) and untreated (E) tumors at 48-hour post ablation time point. F provides the calculated displacement: 6.0 ± 0.3 μm before and 2.9 ± 0.7 μm after ablation.

The displacement was also mapped in NDL tumors *in vivo* (Fig 6) and changes were detected following ablation. The location of the peak displacement was visible through changes in the phase images during the application of radiation force (Fig 6A and 6B) where the red arrow indicates the location of the ultrasound focus. However, no changes were visible on the corresponding magnitude image (Fig 6C). The displacement observed within the tumor was evaluated before and 48 hours after thermal ablation of a 1 mm^3^ region within the center of the tumor. The ablated region was clearly visible on hematoxylin and eosin histology 48 hours after treatment in the treated (Fig 6D) but not control (Fig 6E) tumor. The average displacement was 6.0 ± 0.3 μm before and 2.9 ± 0.7 μm after ablation (Fig 6F). The significant decrease (p < 0.01) in displacement resulted from the increase in the stiffness of the tumor after ablation.

### Shear wave velocity estimation

**Fig 7:**
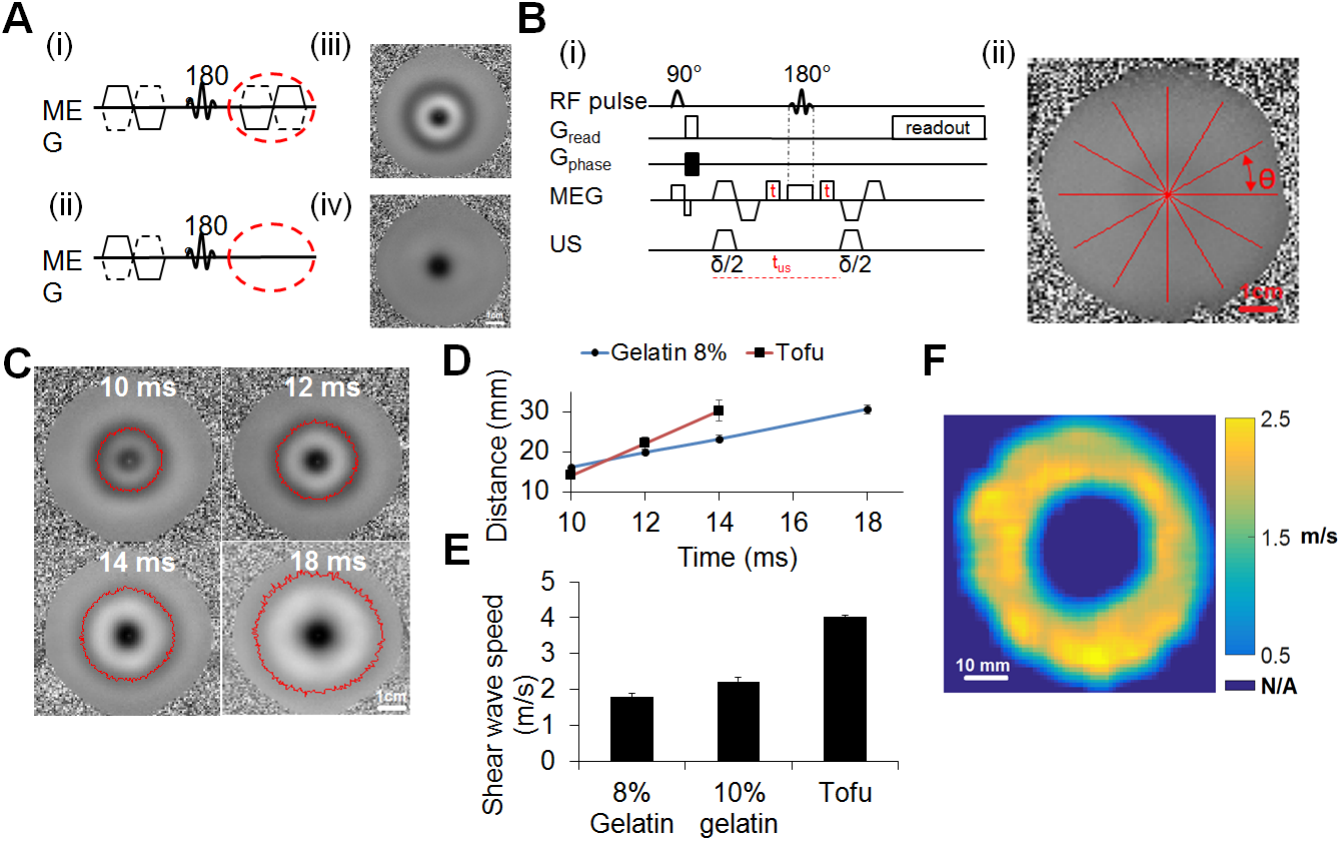
Shear wave visualization. (A) Effect of the second Motion Encoding Gradient (MEG) on the phase images. TE = 23.8 ms, δ = 7 ms, G_e_ = 140 mT/m. The images are 85 × 85 mm^2^ in size. MR sequences (i,ii) and (iii,iv) the corresponding phase images of the shear wave. (B) Shear wave velocity estimation. (i). MR sequence used to estimate shear wave speed by varying delay t. (ii). Shear profiles analysis. Lines are extended from the focus outwards in all directions (360 degree sweep, at θ = 1 degree increment) to determine the distance of the peak minima from the focus. Propagation speed of the shear wave was calculated by measuring the radii of the peak and minima as a function of the delay time. (C) Shear wave propagation in 8% gelatin phantom. Phase images at shear wave propagation times of 10, 12, 14 and 18 ms corresponding to time delays t = 0, 1, 2 and 4 ms. (D) Distance of the peak minima from the focus versus delay time t in 8% gelatin and tofu phantoms. (E) Shear wave velocity in 8, 10% gelatin and tofu phantoms. (F) Shear wave velocity map.

Expanding shear waves were evaluated as an alternative method to visualize the changes in material properties using the spin-echo sequence illustrated in Fig 1. The nature of the expanding shear wave was then validated (Fig 7A). When the second MEG was eliminated, the expanding shear wave was no longer observed (Fig 7A(ii) and Fig 7A(iv)), indicating that the shear wave was produced by the ultrasound during the first MEG, propagated and was captured in the second MEG (Fig 7A(i) and Fig 7A(iii)). To further demonstrate that the expanding ring was due to the shear wave propagation resulting from the first ultrasound excitation, a modified ARFI sequence was tested by varying the delay time t (Fig 7B(i)) demonstrating that the diameter of the expanding shear wave increases with increasing time. By fitting a circle to the expanding wave (Fig 7B(ii)) as a function of the angle, the velocity was calculated in a robust manner. The propagating shear wave was visualized in the gelatin phantom with total propagation times of 10 to 18 ms (delay times of t = 0, 1, 2 and 4 ms) which resulted in radial shear wave travel distances of 14 to 30 mm (Fig 7C and 7D). The STD of the distance measurement also increases with the delay as expected due to the increase in TE (which decreases the SNR) and the decreased amplitude of the shear wave as it propagates. The STD of the shear wave travel distance for the 8% gelatin phantom was 0.6, 0.7, 0.8 and 1.1 mm for the 10, 12, 14 and 18 ms delay, respectively. For measurements in the tofu phantom, the STD of the distance measurements was 0.8, 1.5 and 2.6 mm with a 10, 12 and 14 ms delay, respectively.

The shear wave velocity was estimated as ~1.7 and 2.2 m/s in the 8 and 10% gelatin phantoms, respectively, demonstrating the effective increase in shear wave velocity with stiffness, as expected from [45]. The shear wave velocity was further increased to 4.0 m/s in tofu. Combining the estimates of velocity based on the set of images in Fig 7C, the spatial estimates of shear wave velocity can also be visualized as a spatial map in order to detect local inhomogeneities (Fig 7F).

## Discussion

A rapid, combined MR-ARFI and shear wave velocity estimation protocol has been demonstrated which has potential applications in interventional radiology HIFU applications. The time required can be as small as ~50 s for a 64x64 matrix and one estimate of the shear wave propagation distance. The ability to rapidly localize the FUS beam while simultaneously detecting changes in tissue properties can be exploited during ablative treatments to assess the progression of treatment. While EPI-based sequences have also been explored for MR-ARFI protocols, the utilization of such sequences is significantly more challenging at 7T especially with tissues located in and around the abdomen that are more prone to respiratory motion as well as to EPI distortion due to shim effects [48]. Therefore, we have chosen a modified spin echo sequence with inverted bipolar MEGs.

Future studies may also use such measurements to correct for deviations in the FUS beam path (which may be caused by different acoustic properties of ablated tissue). Here, we combined 7 T MRI and 3 MHz focused ultrasound to improve the MRI spatial resolution and minimize the volume of sonicated tissue. As shown in our study, changes in tissue mechanical response to acoustic radiation force can be detected after ablation. However, such an evaluation is particularly challenging in a small region of interest, such as that evaluated here (~5 mm). In the framework of clinical HIFU ablation, the techniques evaluated here could be of interest for early assessment of thermal ablation due to their larger size.

### MR-ARFI

We have demonstrated that our MRgFUS system could be used for MR-ARFI in both phantoms and *in vivo* studies. In addition, we were able to obtain focal displacement and shear wave velocity estimates with the same MR-ARFI sequence. Thus, changes in tissue properties can be estimated in less than 30 s rather than the several minutes or more required with standard MRE techniques. Moreover, since many HIFU applications are performed under MR guidance, this technique is well suited to several therapeutic applications. Although ultrasound methods for the evaluation of tissue stiffness allow for even faster acquisitions, our method enhances performance in MRgFUS, where the lack of an imaging ultrasound array prevents the rapid ultrasound elastography techniques from being used or in organs such as the brain where no echography is feasible. This technique allows us to localize the beam prior to treatment while concomitantly generating a pre-treatment displacement value against which we can later compare displacement after treatment to assess treatment efficacy.

The measured displacement was proportional to the acoustic intensity. Therefore, the spatial distribution of the beam can be assessed. Using the ARFI sequence, the temperature was not significantly increased during sonication, which is useful in guiding drug delivery, especially when guiding the delivery of temperature-sensitive drugs. A limitation of this study is that the accuracy of the measured FUS induced displacement was not validated. Although not available at our site, one possible method to validate the result is to use an MR-compatible piezoelectric actuator with a predetermined displacement (millimeter-scale).

Respiratory and cardiovascular motion are two primary sources of artifact. Motion of the object resulted in inconsistencies in phase and magnitude, which then result in blurring and ghosting. With the aid of respiratory gating, the image quality was improved and the motion artifact was removed. However, the penalty for applying the respiratory gating technique was the prolonged acquisition time. Possible methods to reduce acquisition time are to use a wider respiratory gating window that brackets expiration, a reduced number of phase encoding steps, or, more interestingly, a navigator based correction of respiratory motion [49]. One can then improve image quality with similar acquisition time as non-gated scans [50].

### Visualization of shear wave propagation

A concurrent MR-ARFI and shear wave velocity estimation protocol has been demonstrated which has potential use in interventional radiology HIFU applications. As with other shear wave-based techniques, an expanding circular wave is easily visualized. We verified recording of the shear wave by removing the secondary MEG (the shear wave generated during the first FUS excitation period propagated to the second MEG period). Furthermore, by changing the delay between the two sonications, the calculated shear wave speed agreed with that given in the literature [45]. This is the first time, to our knowledge, that a single MR sequence was used to visualize and quantify a displacement induced by the acoustic radiation force in addition to providing an estimate of shear wave velocity.

Unfortunately, the expanding shear wave was not observed in the *in vivo* study within the small region of interest in the mouse. We hypothesize that this is due to the small region of interest and the shear wave attenuation, which reduced the amplitude of later waves. As noted in the methods, the calculated shear wave speed in breast fatty tissue is 1.7 m/s, in breast parenchyma is 3.1–3.2 m/s and in invasive ductal carcinoma is 6.6 m/s and the phantoms used here were chosen to mimic these velocities [44]. Here, the tumor is small, ~4 to 5 mm in diameter. Based on previously reported values for shear wave speed, in order to observe shear wave propagation within the tumor, encoding of the shear wavefront in the phase of the signal must be completed in 1 to 2 ms. In our case, however, the time between the two ultrasound excitations was ~6.5 ms, which means that the wavefront of the shear wave reached the tumor boundary and the attenuation of subsequent wavefronts put them below the noise floor. Additional optimization could potentially enhance the shear wave visualization protocol *in vivo*. Parameters, such as the FUS excitation duration and power and the time between MEGs, may be optimized to limit the attenuation of the shear wave within the measured region of interest. By increasing the FUS excitation power or shortening the time between the two MEGs, the initial wave front could be captured and shear wave velocity can be estimated in a relatively small distance, which could facilitate use in small animal studies. Nevertheless, we hypothesize that this limitation is primarily a function of the small size of the tumor model examined here, and would not be a limiting factor in larger animals.

Thus, while we have shown proof of concept for visualization of the shear wave in a phantom with a strategy that also facilitates simultaneous visualization of the FUS-induced displacement at the focus, the method applied here must be modified for a small target volume *in vivo* or applied to larger organs or animal models. Other MRE techniques could also be considered. For instance, a gradient echo phase contrast MRI sequence including an additional MEG could be applied [31]. Alternatively, the ultrasound could be triggered prior to the first motion encoding gradient to improve visualization of the shear wave within a small region of interest, although this would reduce the ARFI signal possibly making beam localization challenging.

In conclusion, we applied MRI methodologies in two ways: evaluating ARFI-based focal displacement and the resulting shear wave speed with a high field MRgFUS system. Focal displacement and shear wave velocity were both obtained with the same MR-ARFI acquisition and the displacement used to detect changes in tissue properties with HIFU treatment. Shear waves were visualized *in vitro* and the velocity estimated rapidly by changing the delay between motion encoding gradients and ultrasound pulses. The sequence described here has potential applications during HIFU treatments to evaluate treatment extent and localize the treatment beam during therapy.
